# Classification of Promoters Based on the Combination of Core Promoter Elements Exhibits Different Histone Modification Patterns

**DOI:** 10.1371/journal.pone.0151917

**Published:** 2016-03-22

**Authors:** Yayoi Natsume-Kitatani, Hiroshi Mamitsuka

**Affiliations:** 1 Japan Science and Technology Agency, PRESTO (Precursory Research for Embryonic Science and Technology), Saitama, Japan; 2 Bioinformatics Center, Institute for Chemical Research, Kyoto University, Kyoto, Japan; Saint Louis University School of Medicine, UNITED STATES

## Abstract

Four different histones (H2A, H2B, H3, and H4; two subunits each) constitute a histone octamer, around which DNA wraps to form histone-DNA complexes called nucleosomes. Amino acid residues in each histone are occasionally modified, resulting in several biological effects, including differential regulation of transcription. Core promoters that encompass the transcription start site have well-conserved DNA motifs, including the initiator (Inr), TATA box, and DPE, which are collectively called the core promoter elements (CPEs). In this study, we systematically studied the associations between the CPEs and histone modifications by integrating the Drosophila Core Promoter Database and time-series ChIP-seq data for histone modifications (H3K4me3, H3K27ac, and H3K27me3) during development in *Drosophila melanogaster* via the modENCODE project. We classified 96 core promoters into four groups based on the presence or absence of the TATA box or DPE, calculated the histone modification ratio at the core promoter region, and transcribed region for each core promoter. We found that the histone modifications in TATA-less groups were static during development and that the core promoters could be clearly divided into three types: i) core promoters with continuous active marks (H3K4me3 and H3K27ac), ii) core promoters with a continuous inactive mark (H3K27me3) and occasional active marks, and iii) core promoters with occasional histone modifications. Linear regression analysis and non-linear regression by random forest showed that the TATA-containing groups included core promoters without histone modifications, for which the measured RNA expression values were not predictable accurately from the histone modification status. DPE-containing groups had a higher relative frequency of H3K27me3 in both the core promoter region and transcribed region. In summary, our analysis showed that there was a systematic link between the existence of the CPEs and the dynamics, frequency and influence on transcriptional activity of histone modifications.

## Introduction

As massively parallel DNA sequencing by next-generation sequencing (NGS) is gaining popularity, biologists have gained better access to genome-wide analysis using NGS-based techniques such as chromatin immunoprecipitation (ChIP)-seq (the combination of ChIP and NGS to determine the locus at which a protein of interest is bound) or RNA-seq (quantitative detection of transcripts by NGS). Moreover, the enhancement of biological databases has been made possible through the efforts of scientists involved in large-scale projects such as the modENCODE project [1]. This project aims to “identify all of the sequence-based functional elements” in model animals, including *Drosophila melanogaster*. For this purpose, researchers have collected comprehensive data, including RNA-seq for transcripts and ChIP-seq for modified histones, under different experimental conditions.

Histones are proteins that function to compact DNA. Histone octamers are formed by two of each of four different histones (H2A, H2B, H3, and H4); DNA wraps around the histone octamer to form a histone-DNA complex called a nucleosome [2]. The biological functions of these histones are mediated by the modification of amino acid residues in the histones. A variety of histone modifications have been reported to date, including lysine acetylation, lysine methylation, arginine methylation, serine phosphorylation, and lysine ubiquitylation [3]. The trimethylation of H3 lysine 4 (H3K4me3) and the acetylation of H3 lysine 27 (H3K27ac) have been well studied as active marks of transcription, while the trimethylation of H3 lysine 27 (H3K27me3) is regarded as an inactive mark [4]. Although the biological functions and molecular mechanisms of these histone modifications have been the focus of many recent investigations, few studies have examined the functions of histone modifications in a dynamic system, such as development.

Many of the recent studies have focused on the core promoter region of RNA polymerase II, which contains the transcription start site (TSS), as some histone modifications, such as H3K4me3, are known to occur around TSSs [4]. The mode of transcription can be grouped into two types: dispersed transcription and focused transcription. In dispersed transcription, multiple TSSs exist in a broad region of about 50–100 nucleotides. In contrast, focused transcription starts at a single TSS or within a narrow region of several nucleotides. Generally, genes that are constitutively expressed generally exhibit dispersed transcription, while genes whose expression is tightly regulated generally exhibit focused transcription. The regulation of focused transcription is thought to be dependent of the structure and function of the core promoter, which varies greatly. The core promoter region is generally defined as the minimal region of DNA required for accurate initiation of transcription by RNA polymerase II. Core promoters contain DNA motifs that are well conserved among species; these motifs are collectively called the core promoter elements (CPEs) [5]. In particular, the initiator (Inr), TATA box, and downstream core promoter element (DPE) are found in focused core promoters and have been studied in detail. The most common Inr (consensus sequence: TCAGTYKNNNTYNR in *D*. *melanogaster* [6]) encompasses the +1 TSS, and other CPEs function cooperatively with the Inr in a distance-dependent manner from the +1 position. The TATA box (consensus sequence: STATAWAAR in *D*. *melanogaster* [6]), whose upstream T is located at -31 or -30 relative to the +1 position in Inr [7], is the most well-studied CPE, although only 28.3% of all core promoters have this element in *D*. *melanogaster* [6]. The DPE (consensus sequence: CRWMGCGWKCGGTTS in *D*. *melanogaster* [6]) functions analogously to the TATA box [8] and is present as frequently as the TATA box [9]. This element is located from +28 to +33 relative to the +1 position in Inr [10]. Studies examining the functions of CPEs have mainly focused on determining which protein(s) will be recruited upon transcription, similar to transcription factor binding sites (TFBSs), and the associations between the CPEs and other regulatory systems, such as histone modification, are not clear. Furthermore, as there are no universal CPEs and CPEs can contain a diverse set of components, the mechanisms through which CPEs regulate focused transcription are thought to be complex and have not yet been elucidated [11].

The purpose of this study was to provide insights into the transcription system regulated by the histone modification status and the combination of CPEs. We hypothesized that basal transcription machinery is dependent on histone modification and the CPEs may function cooperatively; therefore, the patterns of histone modifications may be affected by the specific combination of CPEs. For example, Negre et al. produced a large-scale dataset that detected the status of histone modifications such as H3K4me3, H3K27ac, and H3K27me3 by ChIP-seq to study the dynamics of histone modifications and determined the quantity of each transcript by RNA-seq at several developmental stages from the early embryo stage to the adult stage in *D*. *melanogaster* [4]. By integrating this super-series dataset and the CPE database, we investigated whether core promoters with different combinations of CPEs exhibited different dynamics and whether the roles and frequencies of histone modifications varied according to the CPEs.

## Results

### Histone modification dynamics depended on the specific combination of CPEs

**Fig 1:**
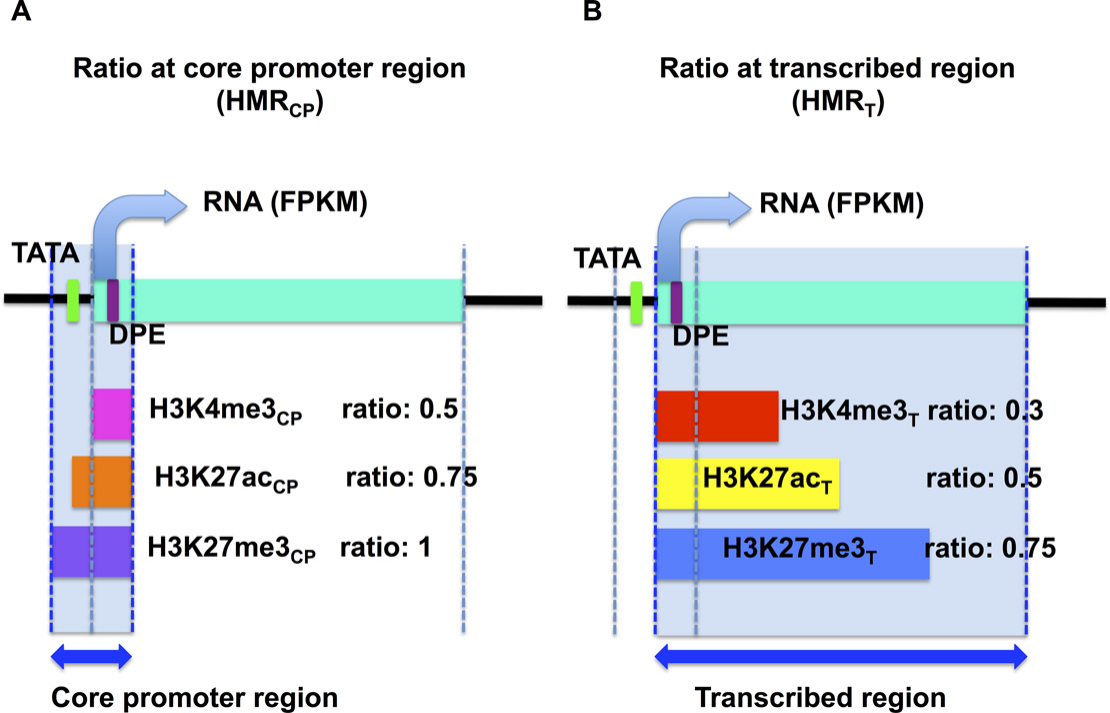
Diagram of histone modification ratios. The green bar represents the transcribed region, and the dotted lines represent the 5′ terminal of the core promoter, +1 position (TSS), 3′ terminal of the core promoter, and transcription end site (TES), from left to right. (A) Histone modification ratios at the core promoter region. The pink bar represents the region with H3K4me3, the orange bar represents the region with H3K27ac, and the purple bar represents the region with H3K27me3 within the core promoter region. The ratios of these bars to the area of the core promoter region filled with light blue were calculated. (B) Histone modification ratios at the transcribed region. The red bar represents the region with H3K4me3, the orange bar represents the region with H3K27ac, and the purple bar represents the region with H3K27me3 within the transcribed region. The ratios of these bars to the area of the core promoter region filled with light blue were calculated.

**Fig 2:**
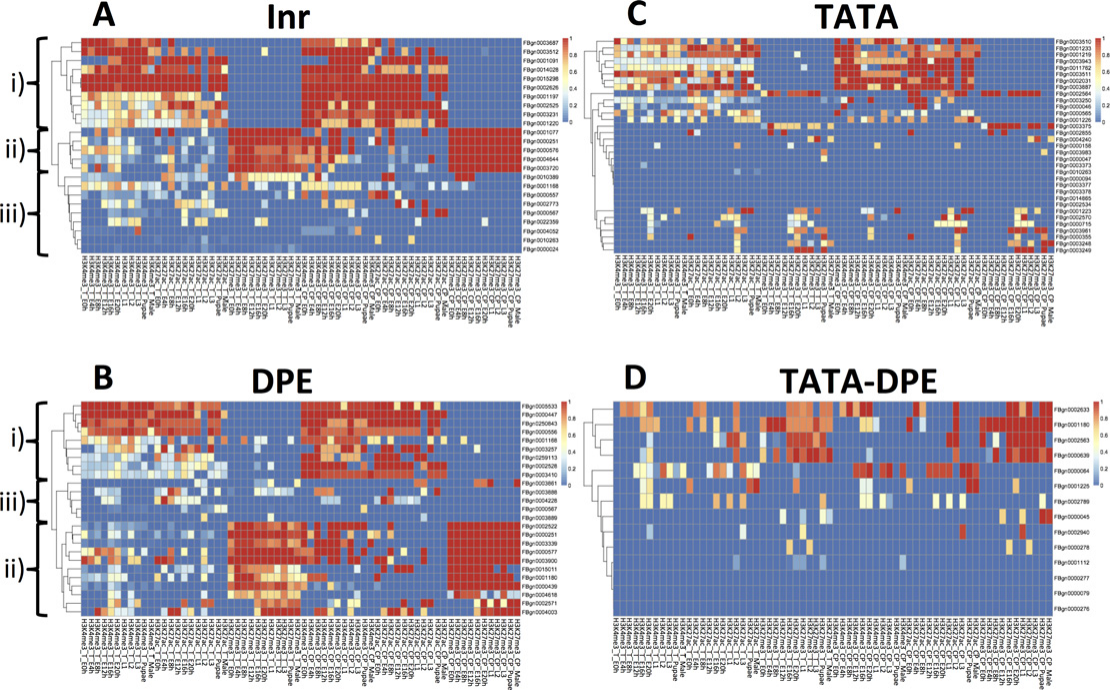
Histone modification dynamics in each CPE group. The y-axis represents genes to which the core promoters were assigned, and the x-axis represents histone modifications (H3K4me3, H3K27ac, and H3K27me3 from left to right for each histone modification) from embryos to adults. E0h: embryo at 0–4 h; E4h: embryo at 4–8 h; E8h: embryo at 8–12 h; E12h: embryo at 12–16 h; E16h: embryo at 16–20 h; E20h: embryo at 20–24 h; L1: larval stage 1; L2: larval stage 2; L3: larval stage 3; Pupae; Male: adult male. The order of genes reflects the results of hierarchical clustering using the pheatmap package in R. The warm color indicates that the histone modification ratio was high. (A) Heatmap of histone modification dynamics in Inr group (n = 24), (B) Heatmap of histone modification dynamics in DPE group (n = 25), (C) Heatmap of histone modification dynamics in TATA group (n = 33), (D) Heatmap of histone modification dynamics in TATA-DPE group (n = 14).

We first examined the sequence-wise overlap between histone modifications and CPEs. We obtained 96 core promoter regions, classified into four types: neither TATA nor DPE (Inr, n = 24), only TATA (TATA, n = 33), only DPE (DPE, n = 25), and both TATA and DPE (TATA-DPE, n = 14). For each core promoter region, we computed the histone modification ratio (HMR) as the ratio of the histone-enriched region (determined by the ChIP-seq dataset in GSE15292) to the entire length of the core promoter (HMR_CP_; Fig 1A). Additionally, we computed the HMR for each transcribed region (HMR_T_; Fig 1B). Fig 2 shows the HMRs according to heatmaps. Obvious differences in HMRs were observed between the TATA-less (i.e., Inr and DPE) and TATA-containing groups (i.e., TATA and TATA-DPE). For the TATA-less groups, core promoter regions were clearly clustered into three types (Inr: empirical *p*-values = 1.49387e-71 and DPE: empirical *p*-values = 4.181458e-66): i) continuously high HMRs in active marks (H3K4me3 and H3K27ac) and continuously low HMRs in inactive marks (H3K27me3), ii) continuously high HMRs in inactive marks (H3K27me3) and few active marks (H3K4me3), and iii) low HMRs (Fig 2A and 2B). The TATA-containing group had a much smaller number of high HMRs, where modifications were unlikely to occur and clear clusters were not found (Fig 2C and 2D).Correlation coefficients between histone modifications and RNA expression values (fragments per kilobase of exon per million fragments mapped [FPKM] obtained from RNA-seq in GSE15292) also showed a clear difference between the TATA-less and TATA-containing groups (S1 Fig). These results implied that there was a relationship between histone modification and CPEs.

### The presence or absence of the TATA box affected the importance of histone modification for transcription

We then performed linear regression analysis to address whether CPEs influenced the role of histone modifications in transcription using a matrix of HMRs. The objective variable for regression was the log transformed FPKM of the sum over all transcripts that shared the TSS of the corresponding core promoter. Among all possible arbitrary combinations, the final regression equation with statistically significant variables is shown below for each dataset, with the correlation coefficient between measured and predicted log(FPKM) and its *p*-value (S2–S5 Figs show diagnostic plots for each dataset):

Inr: *r* = 0.593, *p*-value < 2.2e-16

$$\text{log}(FPKM)=2.98\times ({K27ac}_{T})+1.61\times ({K4me3}_{CP})-0.80\times ({K27me3}_{CP})+0.82$$

DPE: *r* = 0.613, *p*-value < 2.2e-16

$$\text{log}(FPKM)=3.38\times ({K4me3}_{T})+1.66\times ({K27ac}_{T})+0.89$$

TATA: *r* = 0.465, *p*-value < 2.2e-16

$$\text{log}(FPKM)=3.13\times ({K27ac}_{T})+1.62\times ({K4me3}_{CP})-1.19\times ({K27me3}_{CP})+0.86$$

TATA-DPE: *r* = 0.357, *p*-value = 2.16e-07

$$\text{log}(FPKM)=3.54\times ({K27ac}_{T})+3.26\times ({K4me3}_{CP})+0.71$$

**Fig 3:**
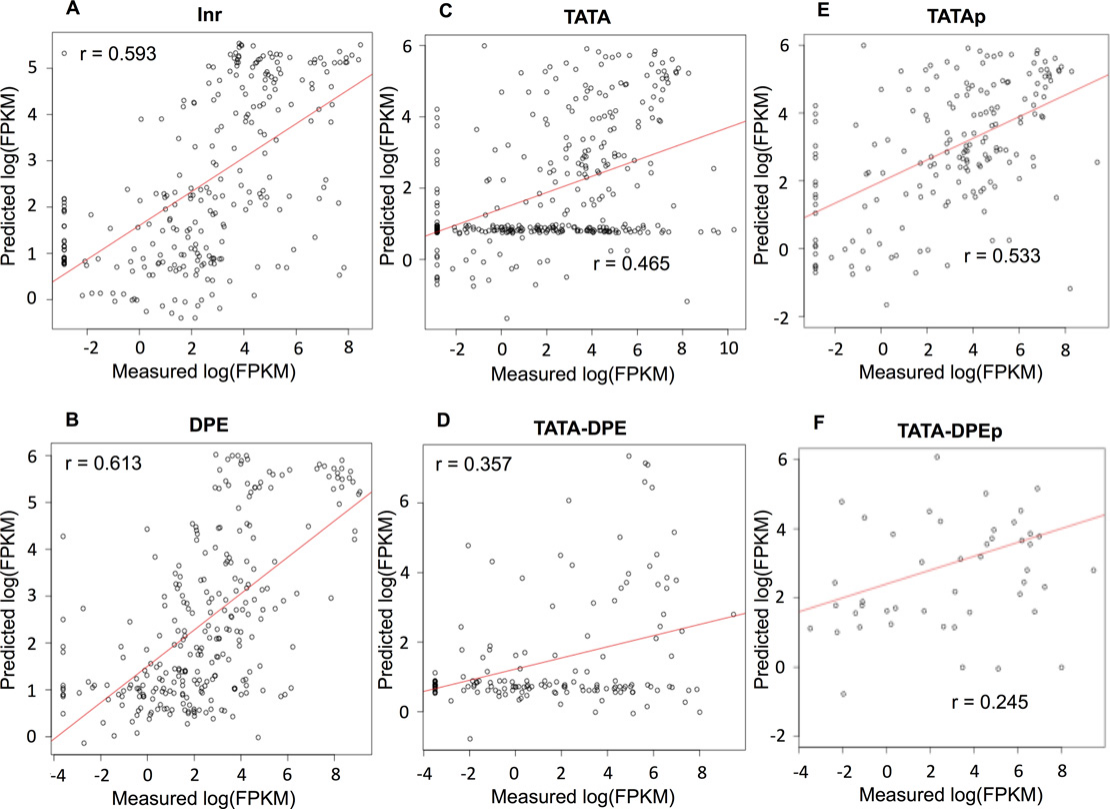
Comparison of the histone modification dependency of RNA expression values by linear regression. Ten-fold cross validation was performed to check that the regression equations reflected the general relationship between the histone modification ratio and the measured log(FPKM) obtained by RNA-seq. The correlation between the measured and predicted log(FPKM) has been represented by a scatterplot. (A) Scatterplot between the measured and predicted log(FPKM) in the Inr group (*n* = (24 *core promoters*) × (11 *developmental stages*) = 264). The correlation coefficient was *r* = 0.593. (B) Scatterplot between the measured and predicted log(FPKM) in the DPE group (*n* = (25 *core promoters*) × (11 *developmental stages*) = 275). The correlation coefficient was *r* = 0.613. (C) Scatterplot between the measured and predicted log(FPKM) in the TATA group (*n* = (33 *core promoters*) × (11 *developmental stages*) = 363). The correlation coefficient was *r* = 0.465. (D) Scatterplot between the measured and predicted log(FPKM) in the TATA-DPE group (*n* = (14 *core promoters*) × (11 *developmental stages*) = 154). The correlation coefficient was *r* = 0.357. The TATA and TATA-DPE groups were divided into two types based on the distributions in the scatterplots. (E) Scatterplot between the measured and predicted log(FPKM) in the TATAp group (n = 189, for details see the Methods). The correlation coefficient was *r* = 0.533. (F) Scatterplot between the measured and predicted log(FPKM) in the TATA-DPEp group (n = 51, for details see the Methods). The correlation coefficient was *r* = 0.245.

All CPE groups, except the DPE group, had similar regression equations, where K27ac_T_ and K4me3_CP_ were positively selected. In DPE-less groups, K27me3_CP_ was negatively selected. Interestingly, TATA-less and TATA-containing groups differed in terms of the predicted log(FPKM) versus the measured log(FPKM) (Fig 3). In the TATA-less groups, a positive correlation was observed between measured and predicted log(FPKM), as we expected (*r* = 0.593 and *r* = 0.613, respectively; Fig 3A and 3B). However, in the TATA-containing groups, the observations could be divided into two types (Fig 3C and 3D): i) TATAp or TATA-DPEp groups, core promoters with a positive correlation between measured and predicted log(FPKM) values (Fig 3E and 3F); and ii) TATAn or TATA-DPEn groups, core promoters with uniformly distributed predicted log(FPKM) versus measured log(FPKM) values (see the Methods section). Theoretically, the predicted values would be equal to the intercept of the equation when zeros were substituted for all the explanatory variables in the regression equation. As we expected, histone modifications occurred in the TATAp and TATA-DPEp groups, but not in the TATAn and TATA-DPEn groups (S6 Fig). These results indicated that the transcription in the TATA-less groups was dependent on histone modification and that the RNA expression levels could be predicted by the histone modification status. In contrast, the TATA-containing groups contained core promoters whose RNA expression values were independent of histone modification status, and a considerable amount of RNA exists, although no histone modifications were detected. We performed linear regression analysis for TATAp and TATA-DPEp and obtained the regression equations below.

TATAp: *r* = 0.533, *p*-value = 2.03e-14

$$\text{log}(FPKM)=2.82\times ({K27ac}_{T})+1.48\times ({K4me3}_{CP})-1.45\times ({K27me3}_{CP})-1.45$$

TATA-DPEp: *r* = 0.245, *p*-value = 0.000819

$$\text{log}(FPKM)=2.91\times ({K27ac}_{T})+2.29\times ({K4me3}_{CP})-1.82\times ({K27me3}_{CP})+2.24$$

Consistent with our previous results, these equations maintained the properties of all CPE groups; K27ac_T_ and K4me3_CP_ were positively selected. In both groups, K27me3_CP_ was negatively selected. The correlation coefficient of TATA was improved from 0.465 to 0.533 by removing TATAn, while this improvement was not observed in the TATA-DPE group (*r* changed from 0.357 to 0.245).To examine the importance of each histone modification for transcription, we determined the relative importance of each explanatory variable in the regression equation using LMG [12]. The results showed that K27ac_T_ was the most influential histone modification on RNA expression levels in all groups except for DPE; K4me3_CP_ was the second most influential modification. Interestingly, K27me3_CP_ was influential in TATAp and TATA-DPEp, regardless of their negative coefficients (Table 1). This result demonstrated that both active and inactive marks influenced transcription equally in the TATA-containing groups, but active marks had more influence than inactive mark on transcription in the TATA-less groups.

**Table 1:** The relative importance of each histone modification in determining RNA expression levels was calculated using the regression equations obtained by linear regression analysis using the LMG method [12]. Values were normalized such that the sum of all values was 1.

Linear regression	Inr	DPE	TATA	TATA-DPE	TATAp	TATA-DPEp
H3K4me3_T_		0.61				
H3K27ac_T_	0.57	0.39	0.57	0.56	0.48	0.37
H3K27me3_T_						
H3K4me3_CP_	0.35		0.37	0.44	0.25	0.28
H3K27ac_CP_						
H3K27me3_CP_	0.08		0.06		0.27	0.35

**Fig 4:**
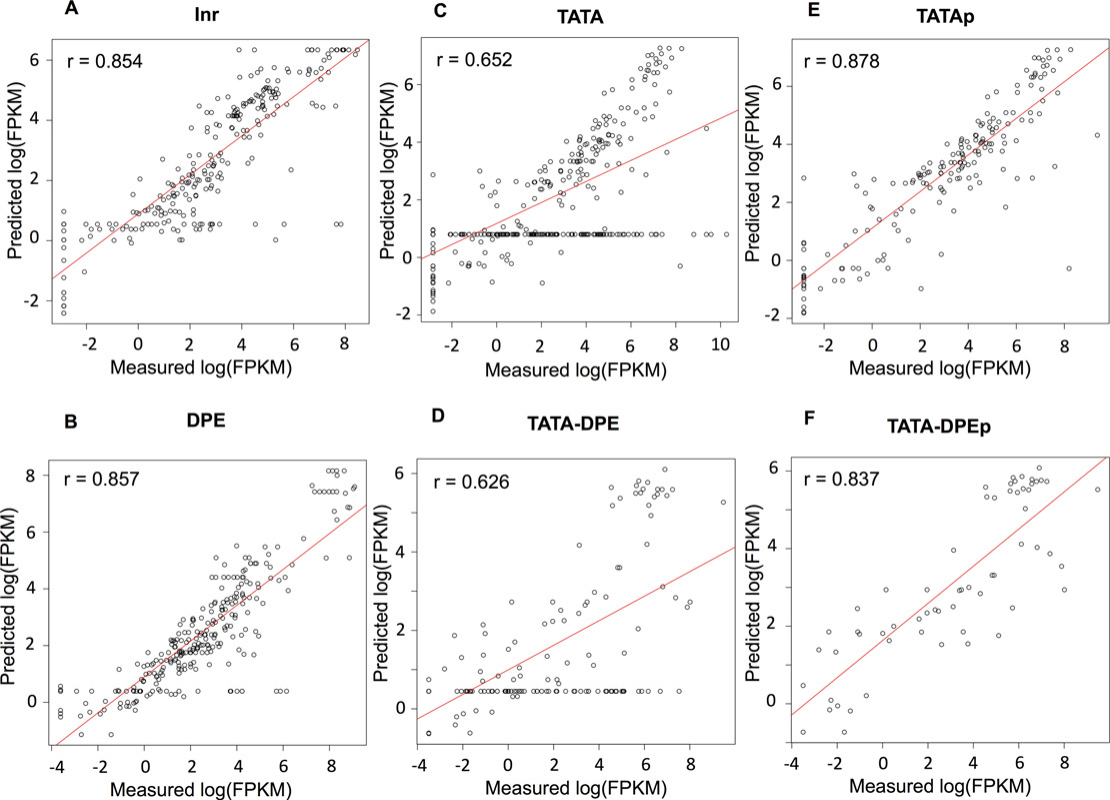
Comparison of the histone modification dependency of RNA expression values by random forest. Non-linear regression by random forest was performed and the correlation between the measured and predicted log(FPKM) has been represented by a scatterplot. (A) Scatterplot between the measured and predicted log(FPKM) in the Inr group (*n* = (24 *core promoters*) × (11 *developmental stages*) = 264). The correlation coefficient was *r* = 0.854. (B) Scatterplot between the measured and predicted log(FPKM) in the DPE group (*n* = (25 *core promoters*) × (11 *developmental stages*) = 275). The correlation coefficient was *r* = 0.857. (C) Scatterplot between the measured and predicted log(FPKM) in the TATA group (*n* = (33 *core promoters*) × (11 *developmental stages*) = 363). The correlation coefficient was *r* = 0.652. (D) Scatterplot between the measured and predicted log(FPKM) in the TATA-DPE group (*n* = (14 *core promoters*) × (11 *developmental stages*) = 154). The correlation coefficient was *r* = 0.626. The TATA and TATA-DPE groups were divided into two types based on the distributions in the scatterplots. (E) Scatterplot between the measured and predicted log(FPKM) in the TATAp group (n = 178, for details see the Methods). The correlation coefficient was *r* = 0.878. (F) Scatterplot between the measured and predicted log(FPKM) in the TATA-DPEp group (n = 46, for details see the Methods). The correlation coefficient was *r* = 0.837.

The results of linear regression showed low linear correlation coefficients between the measured and predicted log(FPKM) for the presence of TATA and the pattern of scatter plot. The measured and predicted values however might have some non-linear correlations, which might result in the unique pattern in Fig 3. In order to validate this hypothesis, we performed random forest, a type of non-linear regression analysis (Fig 4). The correlation coefficients between the measured and predicted log(FPKM) were clearly improved from 0.465 to 0.652 for TATA group by using random forest (from 0.357 to 0.626 for TATA-DPE group). Also core promoters were distributed uniformly in the resultant scatterplot for TATA-containing group by random forest (Fig 4C and 4D), showing that the unique pattern was also obtained by random forest.

We compared the importance of each explanatory variable in random forest (mean decrease in node impurity in Table 2) with the relative importance obtained by linear regression shown in Table 1. The importance by random forest showed a similar trend to the relative importance obtained by linear regression: the importance of H3K27ac_T_ is higher than that of H3K4me3_T_ in all groups except for DPE. Moreover, the importance of H3K27me3_CP_ in TATA-containing groups (i.e., TATAp and TATA-DPEp) was higher than that in TATA-less groups (i.e., Inr and DPE).

**Table 2:** The variable importance of each histone modification in determining RNA expression levels was obtained by random forest as mean decrease in node impurity. The matrices were normalized such that their sum was 1.

Random forest	Inr	DPE	TATA	TATA-DPE	TATAp	TATA-DPEp
H3K4me3_T_	0.21	0.30	0.23	0.15	0.22	0.13
H3K27ac_T_	0.27	0.23	0.29	0.23	0.24	0.19
H3K27me3_T_	0.08	0.11	0.08	0.19	0.12	0.29
H3K4me3_CP_	0.22	0.17	0.15	0.15	0.13	0.08
H3K27ac_CP_	0.18	0.13	0.21	0.15	0.19	0.09
H3K27me3_CP_	0.04	0.05	0.05	0.13	0.09	0.21

### The presence or absence of the DPE motif affected the frequency of H3K27me3

**Fig 5:**
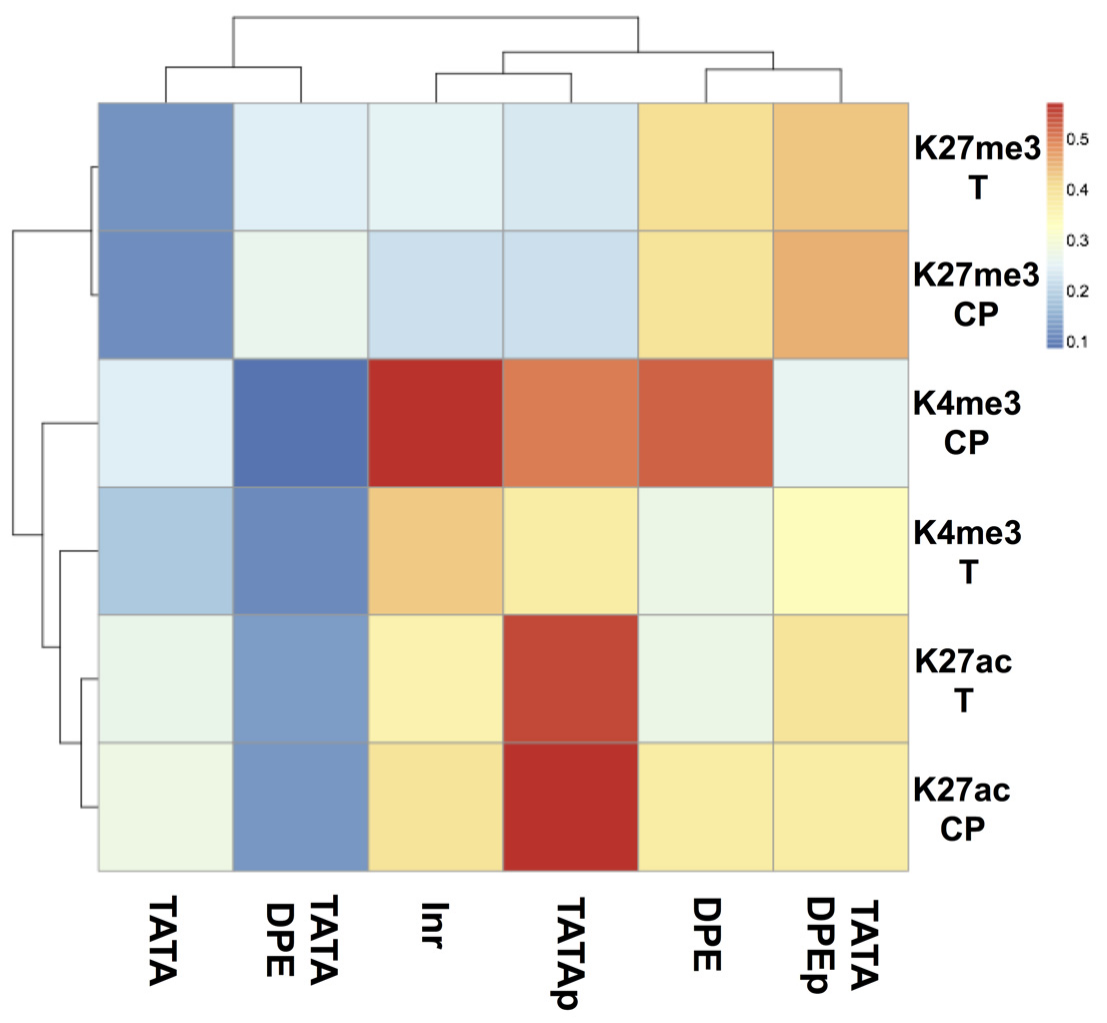
Heatmap of the relative histone modification frequency. The frequency was obtained by calculating the ratio of core promoters whose histone modification ratio was more than 0.5. The y-axis represents the histone modifications, and the x-axis represents the CPE groups. The orders in the y- and x-axes reflect the results of hierarchical clustering using the pheatmap package in R. The warm color represents the high ratio of core promoters whose histone modification ratio is more than 0.5.

We created a heatmap of the ratio of core promoters with HMRs of more than 0.5 to compare the frequencies of histone modifications (Fig 5). As shown in Figs 3–4, the TATA and TATA-DPE groups had lower HMRs because of the TATAn and TATA-DPEn groups, which did not have any histone modifications. We compared the TATAp and TATA-DPEp groups with the other CPEs to determine which histone modifications were relatively frequent. The relative frequency of histone modifications showed CPE-specific patterns (Fig 5). For example, the DPE-containing groups (i.e., DPE and TATA-DPEp) had a higher frequency of H3K27me3 in both the core promoter region and the transcribed region. Notably, both high-frequency histone modifications observed in the DPE-less groups (i.e., K4me3_CP_ and K27ac_T_) influenced transcription according to linear regression analysis.

**Fig 6:**
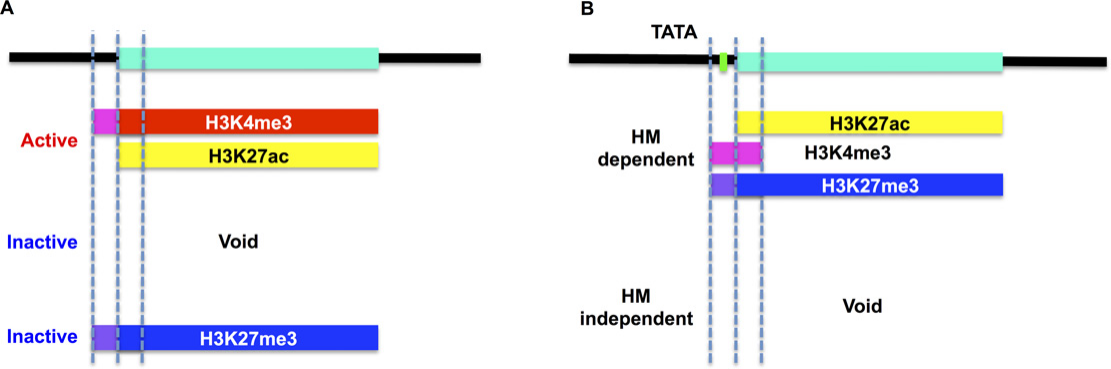
Hypothetical model of the function of the TATA box. The green bar represents the transcribed region, and the dotted lines represent the 5′ terminal of the core promoter, +1 position (TSS), and 3′ terminal of the core promoter, from left to right. (A) Model of TATA-less core promoters. TATA-less core promoters exhibit reduced temporal changes during development and could be grouped into three types: core promoters with continuous active marks, those with occasional histone modifications (void), and those with continuous inactive marks. Because core promoters of the first type do not have inactive marks, their RNA expression values can be predicted by the status of the active marks alone. (B) Model of TATA-containing core promoters. TATA-containing core promoters exhibit increased temporal changes during development and could be grouped into two types: core promoters whose RNA expression values are dependent on the status of histone modifications, and core promoters whose RNA expression values are uniform, despite the void state of histone modifications. Since core promoters of the first type have both of active and inactive marks, all of the histone modification statuses are informative for prediction of their RNA expression values.

In summary, TATA-less core promoters showed small dynamic changes and could be divided into three types: i) continuous with active marks, ii) continuous with inactive marks, and iii) void state and transcriptionally inactive. Since the inactive mark rarely occurred in the first type, the RNA expression values could be predicted by the status of the active marks alone (K27ac_T_ and K4me3_CP_, Fig 6A). In contrast, the TATA-containing core promoters showed substantial dynamic changes and could be divided into two groups: i) those for which RNA expression values were dependent on histone modifications, and ii) those in the void state but for which a considerable amount of RNA existed (Fig 6B).

## Discussion

In this study, we examined the effects of the CPEs and histone modifications on transcription. We found that promoters with different combination of CPEs had different patterns in the dynamics, transcriptional influence, and frequency of histone modifications. Thus, our data provided important insights into the influence of histone modification on RNA expression in the context of specific CPEs.

Prior to our research, several groups have already reported the correlation among histone modification, genomic location, GC content, TF-binding affinities and gene expression profile. Ernst and Kellis developed software called ChromHMM that outputs chromatin state annotation to characterize genome by chromatin state [13]. Benveniste et al reported that histone modifications could be predicted from TF-binding data with high accuracy and suggested that an indirect effect of interactions between TFs and chromatin-modifying enzymes as well as a direct effect of these enzymes could explain the relationship between the pattern of histone modification and gene expression [14]. In addition, DNA methylation as well as histone modification played important roles in TF-binding prediction at location near the TSS [15]. Histone modification is also suggested to be a potential determinant in the prediction of TF-TF co-occupancy (binding regions that are shared between a certain TF and its partner), and the prediction accuracy was improved by the addition of GC content [16]. Dong et al. successfully predicted gene expression values with high accuracy by using both classification and regression of chromatin features [17]. Cheng et al showed that both of TF-binding and histone modification were highly predictive of gene expression profiles and the combination of these two factors didn’t improve the prediction accuracy, which implied their redundancy for gene expression regulation [18]. This tendency was validated by using data from CAGE [19], and it was also demonstrated that expression levels of promoters with high CpG content (HCP genes, according to [20]) were more predictable than those with low CpG content (LCP genes) and different histone modifications were important in these two groups of promoters [17]. This finding is consistent with the report by Mikkelsen et al. in which they demonstrated that promoters with low CpG content were associated with cell type-specific genes and have different histone modification pattern [21]. Interestingly, general or nonspecific TFs were shown to be significantly more predictive than sequence-specific TFs, chromatin structure factors, chromatin remodeling factors, histone methyltransferase and PolIII-associated factors [19]. These reports imply the possibility of the involvement of CPEs that are related with GC contents in promoter regions and binding of general TFs.

Our results are consistent with these reports described above and further suggested that the combination of CPEs was related with how histone modifications were utilized to control temporal change in gene expression. Indeed, the histone modification patterns in the TATA-containing groups were not stable from the early stage of the embryo to the adult stage, implying that deacetylation or demethylation occurred more often in these groups. Kavurma et al. reported that the association of TATA-binding protein (TBP) and phosphorylated p53 and subsequent recruitment of histone deacetylase 1 (HDAC1) mediate the repression of insulin-like growth factor 1 (IGF1R) expression caused by oxidative stress [22]. Direct or indirect protein-protein interactions, such as that between TBP and HDAC1, may alter the histone modification status. However, this remains to be investigated in detail. The TATA box has been shown to be found in genes related to stress responses [23]. In TATA-containing groups, the transcriptional activity of genes involved in the stress response should reflect the extracellular environment, and the dynamics of histone modification may be linked to the flexibility such responses require. Additionally, DPE tends to be found in genes related to development, such as HOX genes [24]. The transcription of these types of genes should reflect the intracellular condition, in which stability (including constitutive repression by H3K27me3) may be more suitable than flexibility. Interestingly, the results of GO analysis showed that genes with the functional categories of “signal” or “secreted” were significantly enriched (false discovery rate [FDR] < 1%) in the TATA-containing groups, while genes with functional categories of “DNA binding”, “developmental protein”, or “nucleus” were significantly enriched in the TATA-less groups (S7 Fig). Our results are highly suggestive of the function of CPEs as selective transcription regulators; specifically, the combination of CPEs enabled the determination of the expression profile for each transcript in cooperation with histone modification regulation to synchronize the expression of genes within similar GO categories.

The characteristic distributions of histone modifications have been studied intensively, and it is reported that H3K4me3 is enriched at sites of transcription initiation [25] and that the H3K27me3 demethylase UTX colocalizes with the elongating form of RNA polymerase II [26]. Interestingly, however, the results obtained by linear regression and random forest suggested that the H3K4me3 ratio at the transcribed region, not at the core promoter region, had strong influence on transcription in the DPE group (Tables 1 and -2). Benayoun et al. reported that broad H3K4me3 domains mark genes that are essential for cell identity and function and are characterized by increased marks of elongation [27]. Since development is a process during with the identity and function of cells is clarified, the regression equation for the DPE group was consistent with this report and suggested that DPE was involved in the regulation of H3K4me3-driven transcriptional elongation.

One of our most exciting findings was the void state in the TATA-containing groups, whose RNA expression values seemed independent of histone modification status. There are three possible explanations for this phenomenon. First, the TATAn and TATA-DPEn groups were not occupied with histones and exhibited TATA-specific transcriptional regulation. Second, these groups may have unmodified histones and exhibit TATA-specific transcriptional regulation. Third, these groups may be in the void state and transcriptionally inactive, despite changes in RNA stability. The first explanation is partly supported by a report showing that the TBP, which is included in basic RNA polymerase II transcription machinery, requires a nucleosome-free region to bind to the core promoter region [28]. We performed k-means clustering of the core promoters according to the histone modification ratio, but this finding in the TATA-containing groups could be detected only by regression analysis (S8 Fig). We will be able to obtain more insights when the experimental data of histone occupancy and RNA stability at each developmental stage in *D*. *melanogaster* are available to compare with the data we used for this analysis. Considering the result of regression analysis in Figs 3 and 4 and the heatmap of the relative frequency of histone modifications in Fig 5, our data suggested that most core promoters in the TATAp group were transcriptionally active. In the TATA-DPEp group, the influential histone modification patterns had lower frequencies, suggesting that core promoters in this group were transcriptionally inactive.

We found that the DPE and TATA-DPEp groups had a higher relative frequency of H3K27me3. Although it is unclear whether this tendency occurs only during development, these data implied that the presence or absence of the DPE sequence affected the histone modification patterns. Models of the relationships between histone modifications and CPEs are shown in Fig 6. Our results implied that H3K27me3 can function to continuously repress transcription at TATA-less core promoters and occasionally repress transcription at TATA-containing core promoters. This concept will be investigated in future studies in our laboratory. We could find the unique relationship between CPEs and histone modification patterns. However the number of promoters we used for this analysis was limited and the global trend remains to be investigated. Possible future work includes genome-wide CPE detection followed by regression analysis using CAGE datasets for detecting TSS [29] and ChIP-seq data for detecting modified histones, obtained from other species or other experimental conditions, which may provide more insights into the generality of the association between histone modifications and CPEs. In this study, the corresponding ChIP-seq data for H3, which we needed in order to examine nucleosome occupancy, was not available for data integration. However, data integration of histone modifications, CPEs, and nucleosome occupancy may reveal whether the TATA-containing core promoters require adjacent nucleosome-free regions for transcription, which does not require histone modification. Several studies suggested that the chromatin structure is dependent on the existence of TATA box [30], and Tirosh I et al reported that genes that occupied proximal-nucleosome (OPN) were highly enriched with TATA boxes compared with genes that depleted proximal-nucleosome (DPN) and that OPN genes showed higher expression variability [31]. To obtain further insights in the association of DNA sequence, histone modification and chromatin structure, we have broadened our interest to integrate not only time-series data but also snapshot data of ChIP-seq or RNA-seq.

## Methods

### Data

All ChIP-seq (GSE16013) and RNA-seq (GSE18068) data are included in GSE15292 [4], obtained by the modENCODE project [1]. We used the ChIP-seq data for H3K4me3, H3K27ac, and H3K27me3 and RNA-seq data obtained at each developmental stage from embryos at 0–4 h to adults.

### Selection of promoters

We obtained DNA sequences of promoters that had only the Inr sequence and no TATA box or DPE sequence (Inr group, n = 64), promoters that had a TATA box but no DPE sequence (TATA group, n = 59), promoters that had a DPE sequence but no TATA box (DPE group, n = 54), and promoters that had both a TATA box and DPE sequence (TATA-DPE group, n = 28) from the Drosophila Core Promoter Database (DCPD, http://labs.biology.ucsd.edu/Kadonaga/DCPD.html) [9]. We confirmed whether these sequences included a TSS according to the current annotation by BLAST in FlyBase [32]. Genes that had several core promoters belonging to different CPE groups were excluded for simplicity. Among them, we selected promoters that did not overlap (Inr: n = 24, TATA: n = 33, DPE: n = 25, TATA-DPE: n = 14).

### Processing of ChIP-seq data for calculation of the histone modification ratio

We downloaded the raw ChIP-seq data in FASTQ format and checked the sequence quality using a FASTX-toolkit (http://hannonlab.cshl.edu/fastx_toolkit/index.html). Sequenced reads were mapped using bowtie-1.0.0 [33]. We built a bowtie-index from BDGP5 using Ensembl and ran bowtie with the parameters–sam–n 3 –m 1. The output files were sorted by samtools-0.1.18 [34] and converted into BED format by bedtools-2.17.0 [35]. For peak detection, we used SICER [36] with an FDR of 1.00e-03, window size of 400 bp for H3K27ac and H3K27me3 and 200 bp for H3K4me3 (considering the tendency of peak width), and gap size of 0 bp (considering the gene density in *D*. *melanogaster*). To calculate the histone modification ratio at the core promoter region, we created a BED format file for each CPE group by submitting the DNA sequences obtained from DCPD to BLAST in FlyBase [32]. For the ratio at the transcribed region, we downloaded the locus information for all mRNAs from the UCSC genome browser (BDGP R5/dm3) [37] in BED format. We defined the transcribed region for each core promoter as the region from the corresponding TSS to the transcription end site (TES) that was the furthest from the TSS among mRNAs whose TSSs were the same. The histone modification ratio was calculated as the overlapping region between the peaks detected by SICER and the core promoter regions or the transcribed regions, as depicted in Fig 1; this information was obtained by bedtools.

### Processing of RNA-seq data for calculation of RNA expression values

After the sequence quality check, carried out as with the ChIP-seq data, the sequenced reads were mapped using TopHat-2.0.8b [38, 39] with the parameters–I 5000 –max-segment-intron 5000 –max-coverage-intron 5000 –bowtie1 –library-type fr-unstranded. We downloaded the index and annotation file (*D*. *melanogaster* Ensembl BDGP5.25) from the TopHat website (http://ccb.jhu.edu/software/tophat/igenomes.shtml) and used this information to run TopHat. To calculate the FPKM for each transcript, Cufflinks-2.1.1 [38, 40–42] was used with parameters–m 100 –s 40 –u–N–I 5000 –library-type fr-unstranded. The FPKM for each core promoter region or transcribed region was calculated as the sum of the FPKMs of transcripts that shared the same TSS. For log transformation, the minimum FPKM in the corresponding CPE group was added to each FPKM in the group to avoid obtaining NA in case the FPKM was equal to zero.

### Visualization

The pheatmap package in R (http://cran.r-project.org/web/packages/pheatmap/index.html) was used to create the heatmaps.

### Calculation of empirical *p*-values for the obtained clusters

First, the squared distance between each sample and its nearest center was summed over all samples as the correct distance. (Pseudo) cluster centers were then randomly generated to compute the distance in the same way as was performed for the correct distance. This random generation (and distance computation) was performed one million times to check whether the correct distance occurred significantly, yielding the empirical *p*-value.

### Linear regression analysis to determine the relative effects of histone modification on transcription

Linear regression analysis was carried out as previously described [43, 44]. We started by creating a scatterplot of the explanatory variables (histone modification ratio) against one another and diagnostic plots to check the homogeneity of variance, the normal distribution of errors, and influential observations (S2–S5 Figs). The leaps package in R (http://cran.r-project.org/web/packages/leaps/index.html) was used for an exhaustive search for the best combination of variables. This returned separate best models of all sizes, and we selected one model whose explanatory variables all significantly affected the objective variance (*p* < 0.05 using the hypergeometric test). In the TATA-DPE group, we selected the model in which one of the explanatory variables (K27me3_TSS_) was not significant (*p*-value = 0.05013), because the relative importance of this variance was reasonably high, as shown in Table 1. We performed 10-fold cross validation and calculated the correlation coefficient between the measured and predicted log(FPKM) values using the bootstrap package in R (http://cran.r-project.org/web/packages/bootstrap/index.html). To obtain the regression equations for the TATAp group, we removed observations in the TATA group for which the predicted log(FPKM) values ranged from 0.7 to 1. The removed observations were treated as in the TATAn group. For the TATA-DPEp group, we removed observations whose predicted log(FPKM) values ranged from 0 to 1. The relative importance of each explanatory variable was calculated by the LMG method, using the relaimpo package in R [45]. The matrices were normalized such that their sum was 1.

### Random forest for non-linear regression to determine the variable importance of histone modification on transcription

The randomForest package in R [46] was used with parameters mtry = 2 and ntree = 500. We used mean decrease in node impurity to evaluate variable importance, which was calculated by the function of the randomForest package. The matrices were normalized such that their sum was 1 for comparison. To obtain the TATAp group, we removed observations in the TATA group for which the predicted log(FPKM) values ranged from 0.7 to 1. For the TATA-DPEp group, we removed observations whose predicted log(FPKM) values ranged from 0 to 1.

## Supporting Information

S1 Fig Comparison of the correlation coefficients between RNA expression values and histone modification ratios. The y-axis represents histone modification ratios, and the x-axis represents the CPE groups. Their orders reflect the results of hierarchical clustering by the pheatmap package in R. The correlation coefficients between RNA expression values and histone modification ratios were visualized by heatmap.
(PDF)

S2 Fig Diagnostic plots in the Inr group. (A) Scatterplot showing correlations among histone modifications. (B) A scatterplot was used to check the homogeneity of variance. (C) The homogeneity of variance was checked using a scale different from that used in (B). (D) A normal Q-Q plot was used to check the normal distribution of errors. (E) Cook’s distance was used to identify influential observations.
(PDF)

S3 Fig Diagnostic plots in the DPE group. (A) A scatterplot was used to show correlations among histone modifications. (B) A scatterplot was used to check the homogeneity of variance. (C) The homogeneity of variance was checked using a scale different from that in (B). (D) A normal Q-Q plot was used to check the normal distribution of errors. (E) Cook’s distance was used to identify influential observations.
(PDF)

S4 Fig Diagnostic plots in the TATA group. (A) Scatterplot showing the correlation among histone modifications. (B) A scatterplot was used to check the homogeneity of variance. (C) The homogeneity of variance was checked using a scale different from that used in (B). (D) A normal Q-Q plot was used to check the normal distribution of errors. (E) Cook’s distance was used to identify influential observations.
(PDF)

S5 Fig Diagnostic plots in the TATA-DPE group. (A) Scatterplot showing correlations among histone modifications. (B) A scatterplot was used to check the homogeneity of variance. (C) The homogeneity of variance was checked using a scale different from that used in (B). (D) A normal Q-Q plot was used to check the normal distribution of errors. (E) Cook’s distance was used to identify influential observations.
(PDF)

S6 Fig Comparison of histone modification ratios among TATAp/n and TATA-DPEp/n groups. The y-axis represents the histone modification ratio, and the x-axis represents histone modifications. (A) Boxplot of histone modification ratios in the TATAp group (n = 189). (B) Boxplot of histone modification ratios in the TATA-DPEp group (n = 51). (C) Boxplot of histone modification ratios in the TATAn group (n = 174). (C) Boxplot of histone modification ratios in the TATA-DPEn group (n = 103).
(PDF)

S7 Fig Enriched functional categories in each CPE group. GO analysis was performed using DAVID Bioinformatics Resources 6.7. The enriched functional categories (SP_PIR_KEYWORDS) with FDRs of less than 1% were selected.
(PDF)

S8 Fig Clustering analysis of histone modification ratios in each CPE group. The k-means clustering was performed with 1000 iteration to obtain five clusters for each CPE group. The distribution of the histone modification ratios in each cluster was visualized by boxplot. The y-axis represents the histone modification ratio, and the x-axis represents the histone modification. The clusters were sorted from top to bottom according to their numbers of core promoters. Bold lines represent clusters in which the medians of histone modifications were equal to zero.
(PDF)
