# Supplementary material for: Classification of Promoters Based on the Combination of Core Promoter Elements Exhibits Different Histone Modification Patterns
Source: PLoS One. 2016 Mar 22;11(3):e0151917. doi: 10.1371/journal.pone.0151917 (PMC4803293; doi:10.1371/journal.pone.0151917)
Supplement: S1 Fig — The y-axis represents histone modification ratios, and the x-axis represents the CPE groups. Their orders reflect the results of hierarchical clustering by the pheatmap package in R. The correlation coefficients between RNA expression values and histone modification ratios were visualized by heatmap. (PDF) [file pone.0151917.s001.pdf]

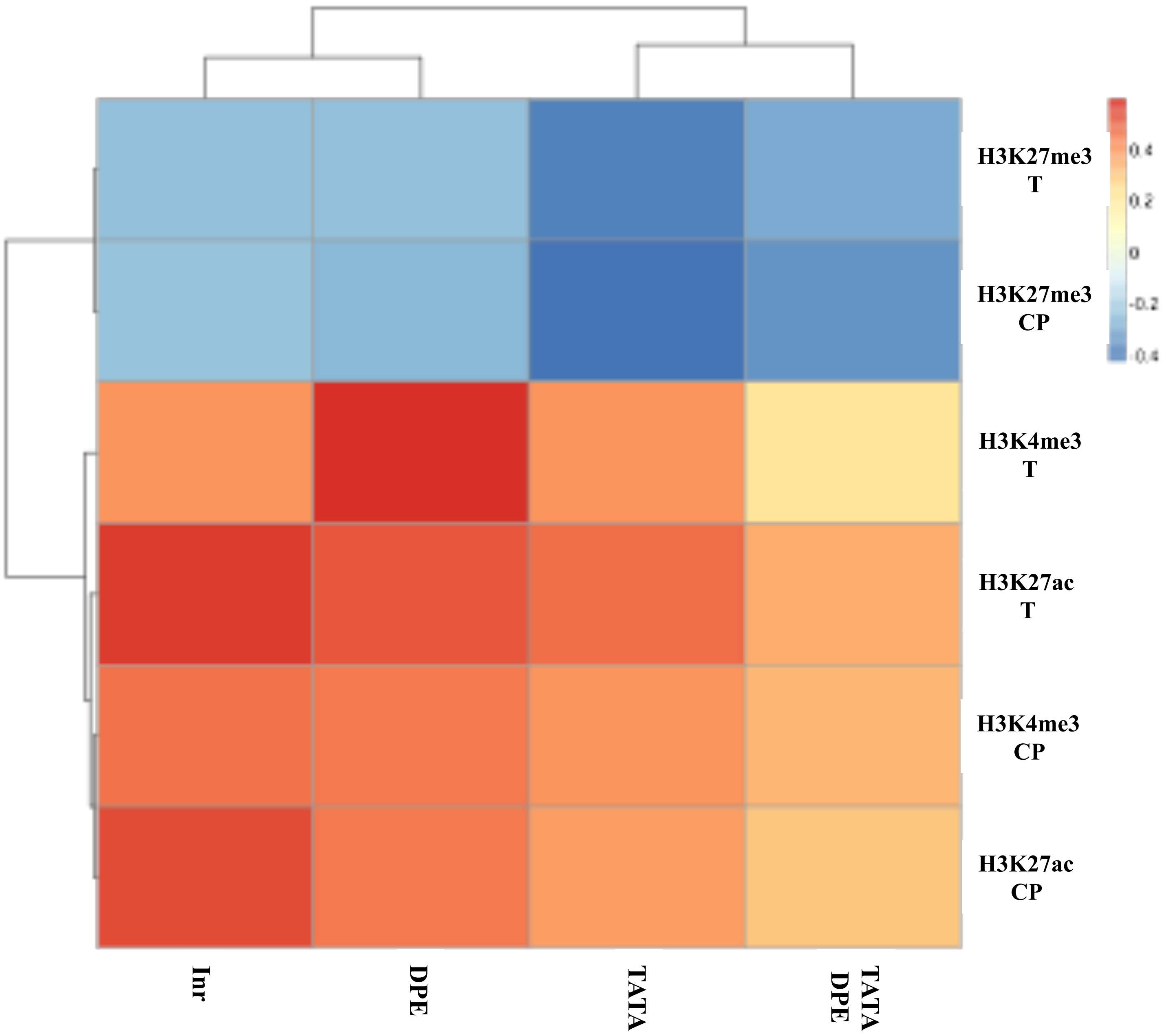

S1 Fig. Comparison of the correlation coefficients between RNA expression values and histone modification ratios.
