# Supplementary figures and images for: Classification of Promoters Based on the Combination of Core Promoter Elements Exhibits Different Histone Modification Patterns
Source: PLoS One. 2016 Mar 22;11(3):e0151917. doi: 10.1371/journal.pone.0151917 (PMC4803293; doi:10.1371/journal.pone.0151917)

A

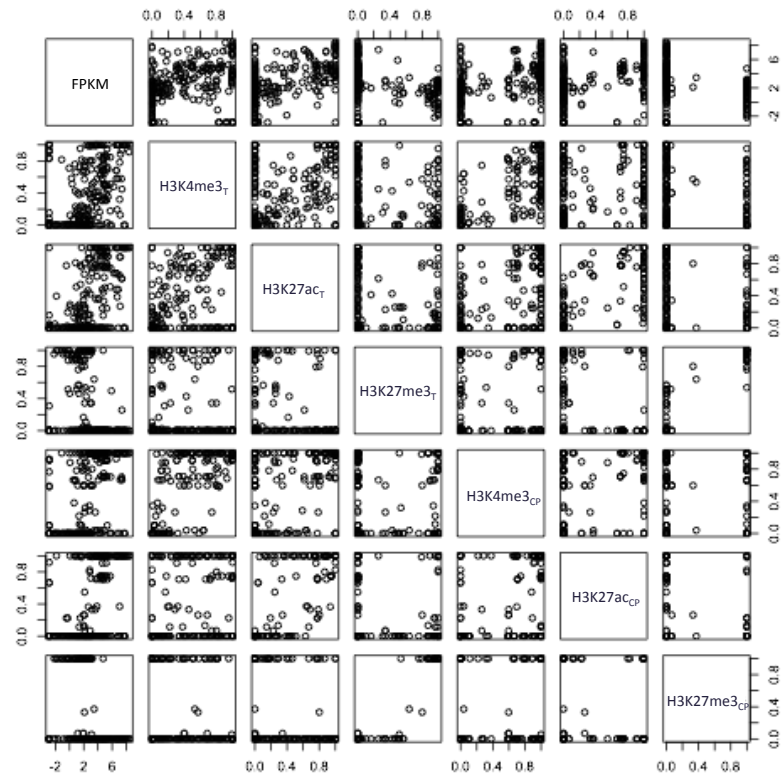

B

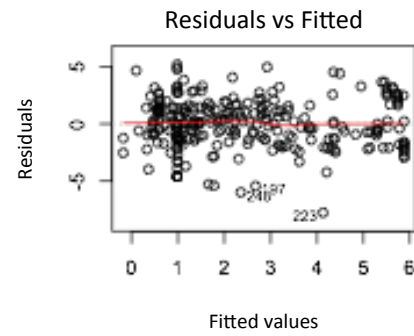

D

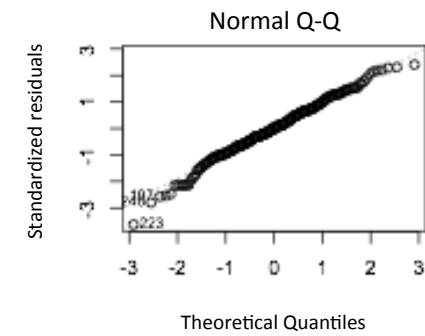

C

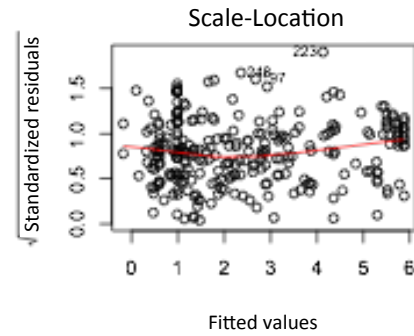

E

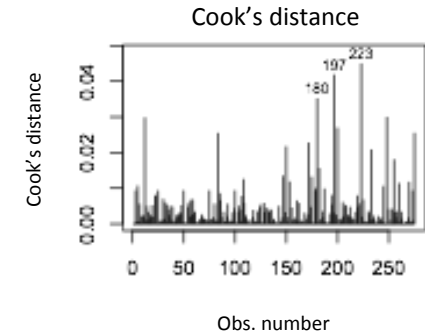

S2 Fig. Diagnostic plots in the Inr group.

Supplement: S2 Fig — (A) Scatterplot showing correlations among histone modifications. (B) A scatterplot was used to check the homogeneity of variance. (C) The homogeneity of variance was checked using a scale different from that used in (B). (D) A normal Q-Q plot was used to check the normal distribution of errors. (E) Cook’s distance was used to identify influential observations. (PDF) [file pone.0151917.s002.pdf]

A

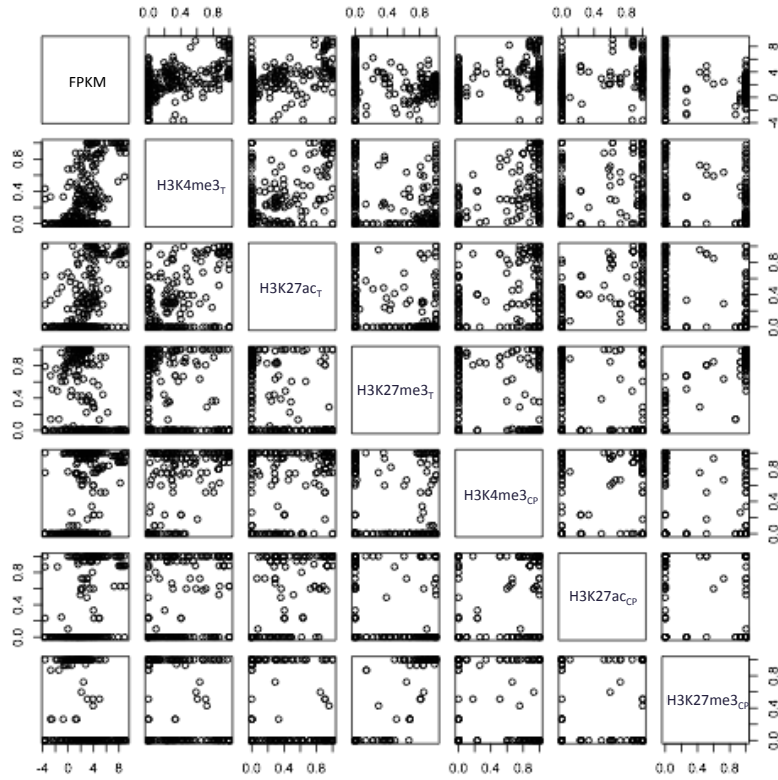

B

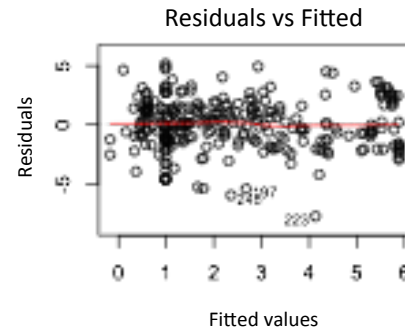

D

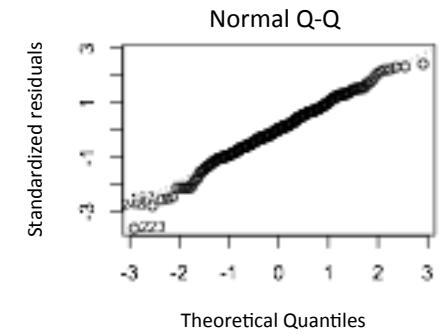

C

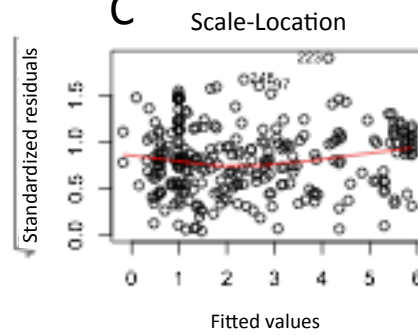

E

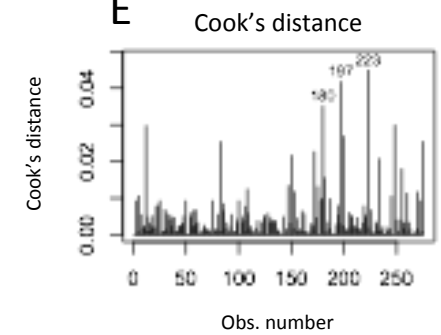

**S3 Fig. Diagnostic plots in the DPE group.**

Supplement: S3 Fig — (A) A scatterplot was used to show correlations among histone modifications. (B) A scatterplot was used to check the homogeneity of variance. (C) The homogeneity of variance was checked using a scale different from that in (B). (D) A normal Q-Q plot was used to check the normal distribution of errors. (E) Cook’s distance was used to identify influential observations. (PDF) [file pone.0151917.s003.pdf]

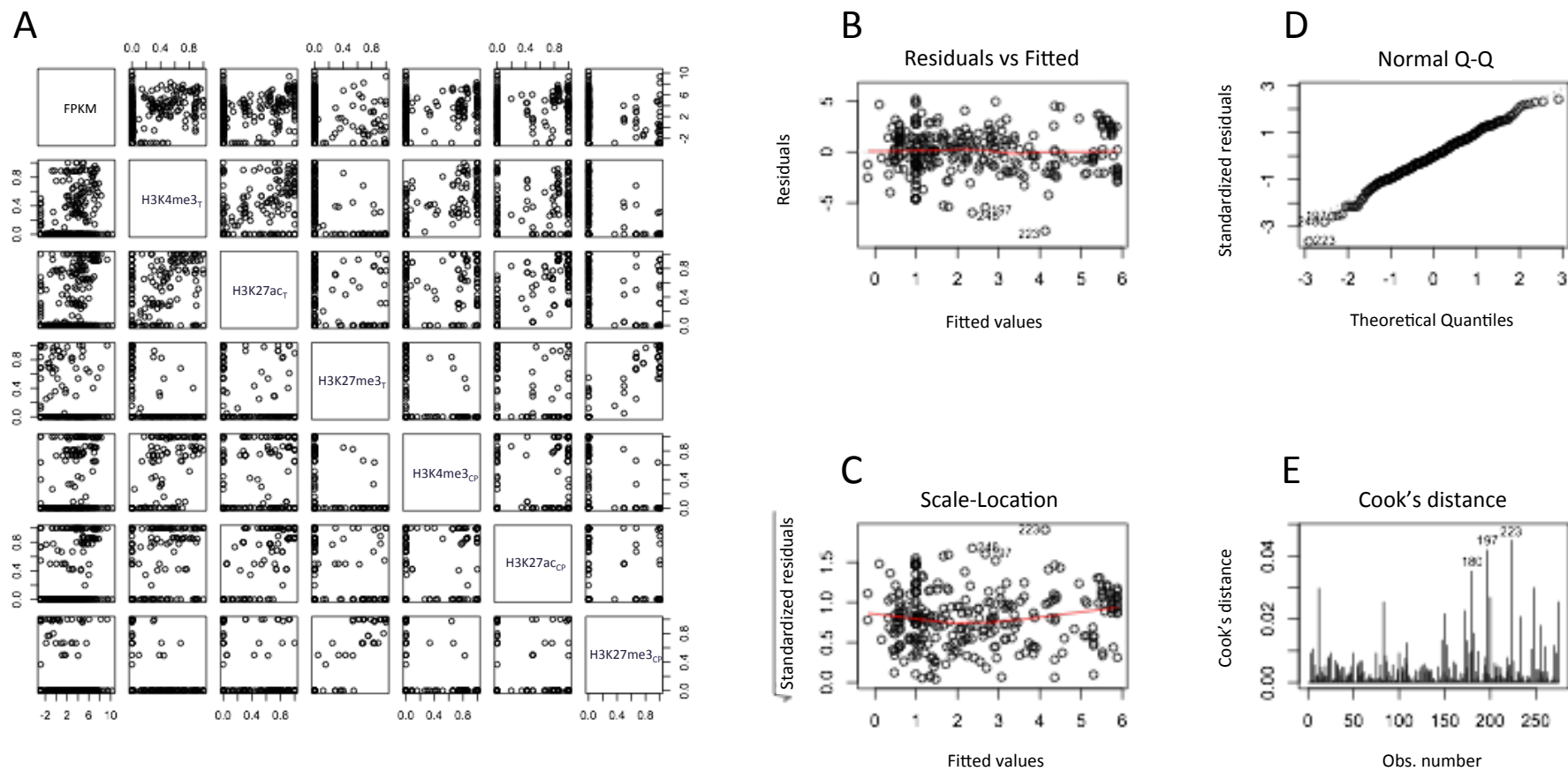

**S4 Fig. Diagnostic plots in the TATA group.**

Supplement: S4 Fig — (A) Scatterplot showing the correlation among histone modifications. (B) A scatterplot was used to check the homogeneity of variance. (C) The homogeneity of variance was checked using a scale different from that used in (B). (D) A normal Q-Q plot was used to check the normal distribution of errors. (E) Cook’s distance was used to identify influential observations. (PDF) [file pone.0151917.s004.pdf]

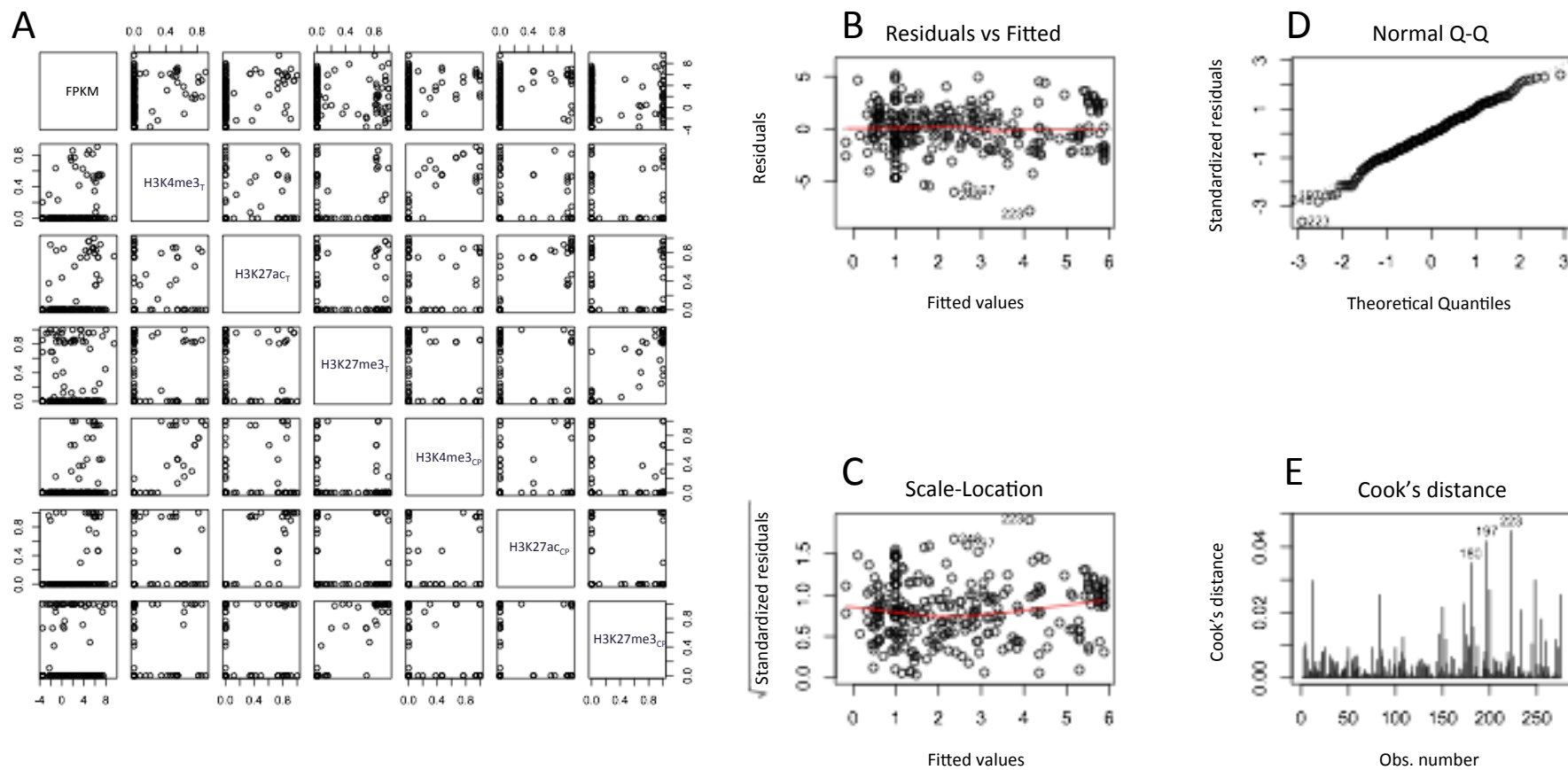

**S5 Fig. Diagnostic plots in the TATA-DPE group.**

Supplement: S5 Fig — (A) Scatterplot showing correlations among histone modifications. (B) A scatterplot was used to check the homogeneity of variance. (C) The homogeneity of variance was checked using a scale different from that used in (B). (D) A normal Q-Q plot was used to check the normal distribution of errors. (E) Cook’s distance was used to identify influential observations. (PDF) [file pone.0151917.s005.pdf]

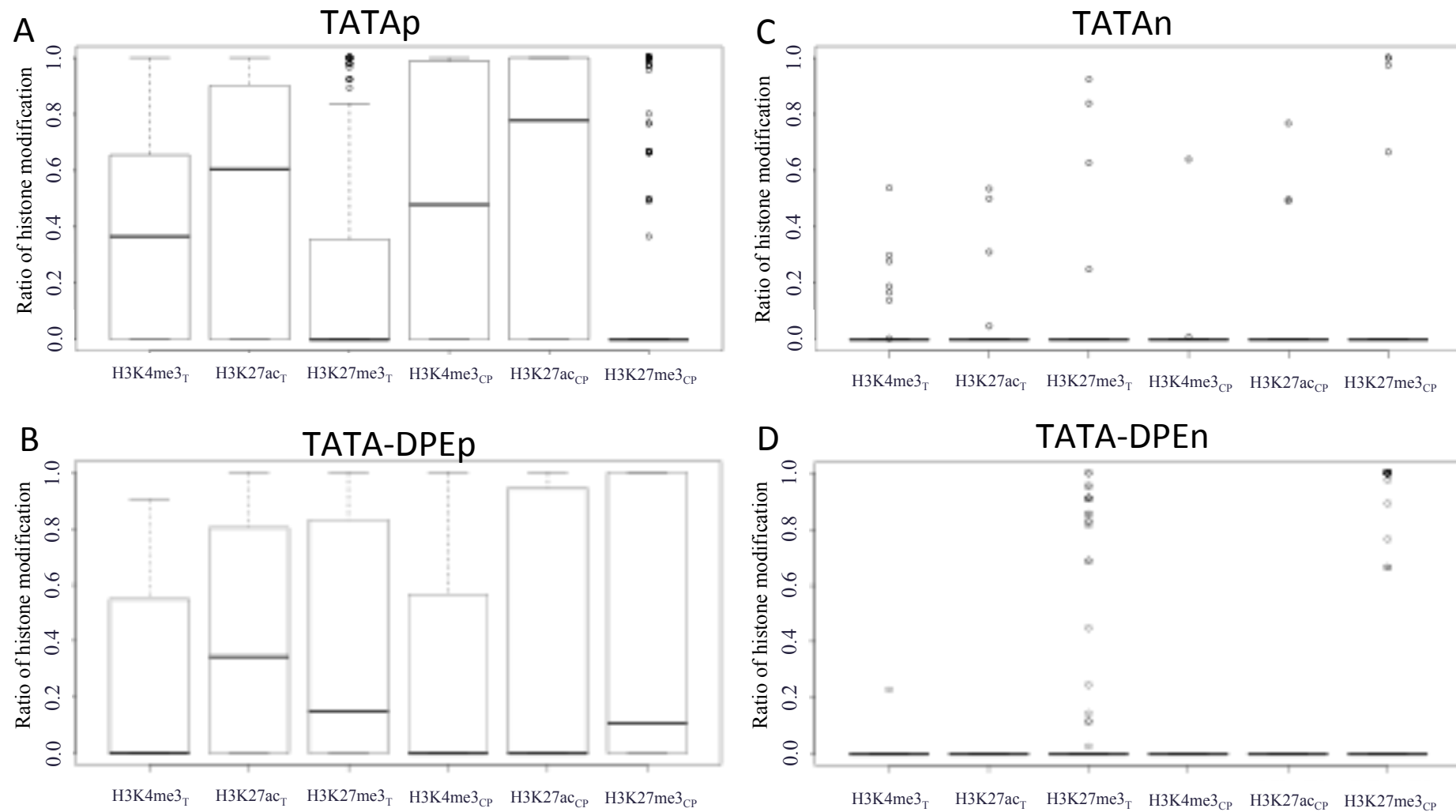

**S6 Fig. Comparison of histone modification ratios among TATAp/n and TATA-DPEp/n groups.**

Supplement: S6 Fig — The y-axis represents the histone modification ratio, and the x-axis represents histone modifications. (A) Boxplot of histone modification ratios in the TATAp group (n = 189). (B) Boxplot of histone modification ratios in the TATA-DPEp group (n = 51). (C) Boxplot of histone modification ratios in the TATAn group (n = 174). (C) Boxplot of histone modification ratios in the TATA-DPEn group (n = 103). (PDF) [file pone.0151917.s006.pdf]

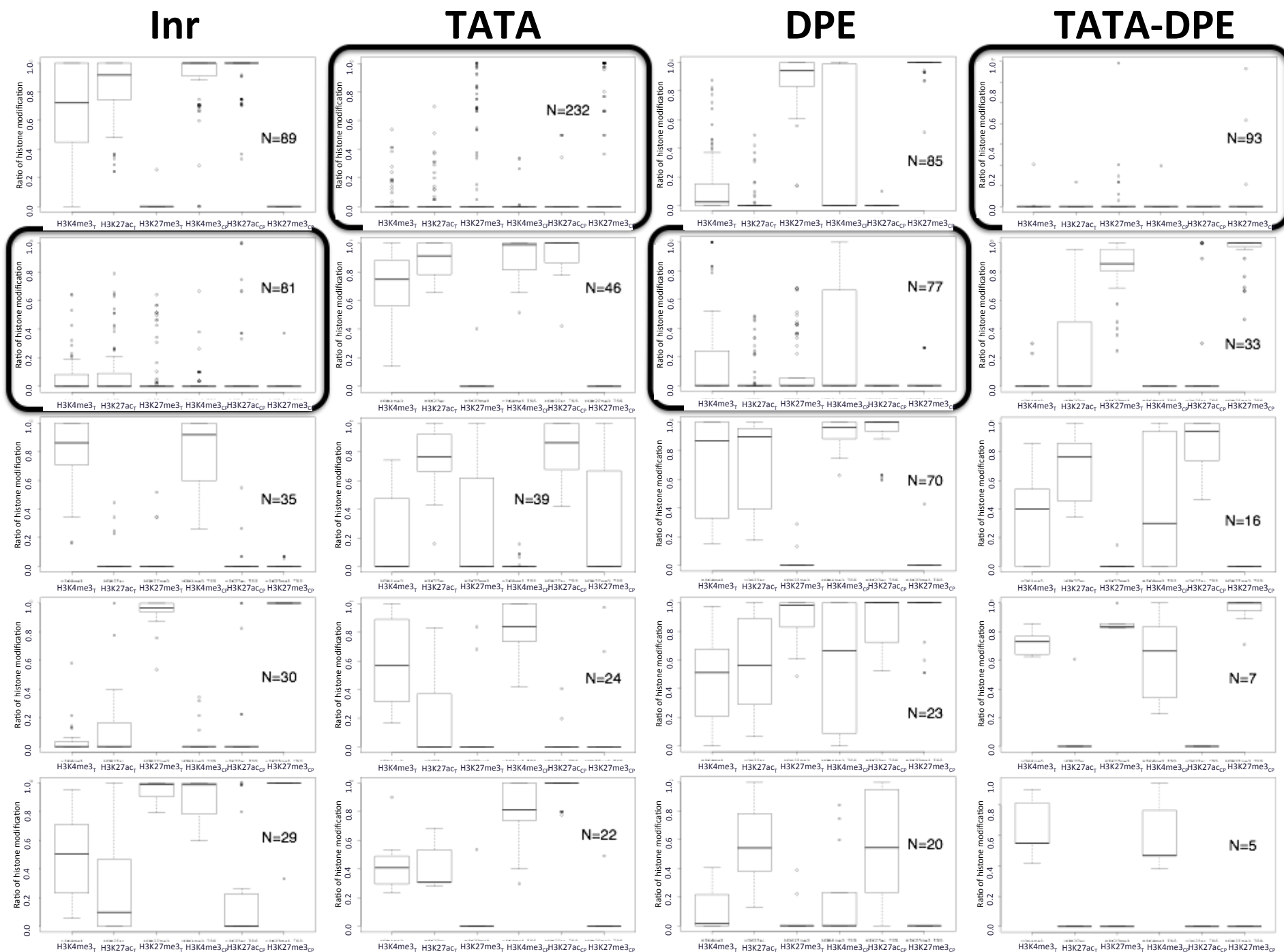

**S8 Fig. Clustering analysis of histone modification ratios in each CPE group.**

Supplement: S8 Fig — The k-means clustering was performed with 1000 iteration to obtain five clusters for each CPE group. The distribution of the histone modification ratios in each cluster was visualized by boxplot. The y-axis represents the histone modification ratio, and the x-axis represents the histone modification. The clusters were sorted from top to bottom according to their numbers of core promoters. Bold lines represent clusters in which the medians of histone modifications were equal to zero. (PDF) [file pone.0151917.s008.pdf]
