# Supplementary material for: Classification of Promoters Based on the Combination of Core Promoter Elements Exhibits Different Histone Modification Patterns
Source: PLoS One. 2016 Mar 22;11(3):e0151917. doi: 10.1371/journal.pone.0151917 (PMC4803293; doi:10.1371/journal.pone.0151917)
Supplement: S7 Fig — GO analysis was performed using DAVID Bioinformatics Resources 6.7. The enriched functional categories (SP_PIR_KEYWORDS) with FDRs of less than 1% were selected. (PDF) [file pone.0151917.s007.pdf]

Inr

Term	Count	%	PValue	Fold Enrichment	Bonferroni	Benjamini	FDR
dna-binding	10	41.7	1.15E-08	13.5	1.04E-06	1.04E-06	1.25E-05
nucleus	12	50.0	4.05E-08	7.8	3.69E-06	1.84E-06	4.42E-05
DNA binding	7	29.2	4.22E-08	32.1	3.84E-06	1.28E-06	4.60E-05
developmental protein	10	41.7	1.07E-07	10.5	9.74E-06	2.43E-06	1.17E-04
phosphoprotein	9	37.5	3.39E-05	6.2	3.08E-03	6.16E-04	3.69E-02
Transcription	7	29.2	3.66E-05	10.1	3.33E-03	5.56E-04	3.99E-02
transcription regulation	6	25.0	3.98E-04	8.7	3.55E-02	5.16E-03	4.33E-01

DPE

Term	Count	%	PValue	Fold Enrichment	Bonferroni	Benjamini	FDR
DNA binding	10	40.0	5.31E-13	42.2	4.94E-11	4.94E-11	5.82E-10
developmental protein	12	48.0	8.91E-10	11.5	8.29E-08	4.14E-08	9.76E-07
dna-binding	11	44.0	1.38E-09	13.7	1.28E-07	4.28E-08	1.51E-06
nucleus	13	52.0	9.61E-09	7.8	8.94E-07	2.23E-07	1.05E-05
transcription regulation	9	36.0	2.81E-07	12.1	2.61E-05	5.23E-06	3.08E-04
Transcription	8	32.0	4.90E-06	10.6	4.56E-04	7.60E-05	5.36E-03
Homeobox	5	20.0	3.65E-05	25.0	3.39E-03	4.85E-04	4.00E-02
phosphoprotein	8	32.0	5.05E-04	5.1	4.59E-02	5.85E-03	5.51E-01

TATA

Term	Count	%	PValue	Fold Enrichment	Bonferroni	Benjamini	FDR
signal	14	42.4	5.00E-12	13.4	4.20E-10	4.20E-10	5.37E-09
Secreted	9	27.3	2.53E-08	17.2	2.13E-06	1.06E-06	2.72E-05
stress response	4	12.1	2.12E-05	70.5	1.78E-03	5.92E-04	2.27E-02
salivary gland	3	9.1	5.50E-05	243.4	4.61E-03	1.15E-03	5.90E-02
retinal protein	3	9.1	8.23E-05	202.8	6.89E-03	1.38E-03	8.83E-02
photoreceptor	3	9.1	1.15E-04	173.8	9.62E-03	1.61E-03	1.23E-01
photoreceptor protein	3	9.1	1.53E-04	152.1	1.28E-02	1.84E-03	1.64E-01
chromophore	3	9.1	1.53E-04	152.1	1.28E-02	1.84E-03	1.64E-01
stress-induced protein	3	9.1	1.53E-04	152.1	1.28E-02	1.84E-03	1.64E-01
heat shock	3	9.1	1.53E-04	152.1	1.28E-02	1.84E-03	1.64E-01
G protein-coupled receptor	3	9.1	2.45E-04	121.7	2.04E-02	2.57E-03	2.63E-01
antibiotic	3	9.1	4.24E-04	93.6	3.50E-02	3.95E-03	4.54E-01
glycoprotein	7	21.2	4.94E-04	6.5	4.07E-02	4.14E-03	5.29E-01

TATA DPE

Term	Count	%	PValue	Fold Enrichment	Bonferroni	Benjamini	FDR
signal	6	46.2	2.41E-05	14.1	1.73E-03	1.73E-03	2.50E-02
polymorphism	4	30.8	1.56E-04	33.6	1.11E-02	5.59E-03	1.62E-01
Secreted	4	30.8	8.48E-04	18.8	5.92E-02	2.01E-02	8.79E-01

S7 Fig. Enriched functional categories in each CPE group.
